# Characterization of the Lipid Oxidation Process of Robusta Green Coffee Beans and Shelf Life Prediction during Accelerated Storage

**DOI:** 10.3390/molecules25051157

**Published:** 2020-03-05

**Authors:** Sha Cong, Wenjiang Dong, Jianping Zhao, Rongsuo Hu, Yuzhou Long, Xiaoxing Chi

**Affiliations:** 1 College of Food Science, Heilongjiang Bayi Agricultural University, Daqing, Heilongjiang 163319, China; congsha1995@163.com; 2 Spice and Beverage Research Institute, Chinese Academy of Tropical Agricultural Sciences, Wanning, Hainan 571533, China; hnhrs@126.com (R.H.); longyuzhou6090@126.com (Y.L.); 3 Tropical Crops Genetic Resources Institute, Chinese Academy of Tropical Agricultural Sciences, Haikou 571700, China; zjp-68068@163.com

**Keywords:** green coffee beans, lipid oxidation, accelerated storage, shelf life prediction

## Abstract

The lipid oxidation process of Robusta green coffee beans was characterized during accelerated storage for 20 days at 40 °C, 50 °C, and 60 °C. The conventional oxidation indexes and fatty acid compositions were evaluated, and the shelf life of the green coffee beans was predicted using the Arrhenius model. The acid value, iodine value, peroxide value, total oxidation value, thiobarbituric acid reactive substances, and free fatty acid content increased throughout storage, while the moisture content, *p*-anisidine value, and unsaturated fatty acid content decreased, which suggests that lipid oxidation occurred during accelerated storage. The predicted shelf life of green coffee bean samples were 57.39 days, 44.44 days, and 23.12 days when stored at 40 °C, 50 °C, and 60 °C, respectively. This study provided scientific evidence of the impact of lipid oxidation on the loss of quality during the accelerated storage of green coffee beans.

## 1. Introduction

Coffee, from the Rubiaceae family, is one of the most popular beverages and an important commercial commodity worldwide. In 2015–2016, the total global coffee consumption was ~9 million tons with a value of close to US$ 21 billion [1], In recent years, the demand and consumption of coffee have increased significantly, mainly because of its unique flavour and other desirable consumption effects. Presently, more than 80% of the world’s adult population consumes coffee beverages [2]. Most green coffee beans are produced from two major species: Robusta (*Coffea canephora* L.) and Arabica (*Coffea arabica* L.). Previous studies have reported that Robusta has an earthy, spicy roast aroma, while Arabica has a sweet, caramel roast aroma [3,4]. In China, coffee is mainly cultivated in Hainan and Yunnan provinces, where both the Robusta and Arabica species are grown, respectively. Xinglong coffee is one of the most popular coffees of China and is protected by the Chines AQSIQ (the People’s Republic of the China’s FDA) “Controlled Designation of Origin” [5]. To prevent quality deterioration during storage and transport, coffee cherries and dried green coffee beans (water content < 11%) undergo pre-processing operations to inhibit enzymatic reactions, microbial spoilage, and lipid oxidation. 

Coffee has attracted much attention since it was revealed to play an important role in nutrition and health, whereby the regular consumption of coffee has been linked to thermogenic effects, the reduction of oxidative stress, the modulation of immune cell function, as well as its usefulness in the treatment of certain illness, such as diabetes mellitus, cardiovascular diseases, and Alzheimer’s disease. In addition, various studies have suggested that the caffeine, chlorogenic acid, fatty acid, vitamin, trigonelline, protein, and lipid contents of coffee may contribute to the abovementioned effects [6,7]. Green coffee beans contain a high proportion of unsaturated fatty acids (USFA) and a low moisture content (MC) and are thus susceptible to lipid oxidation and rancidity during storage. These changes not only produce rancid odours, but also influence the nutritional quality and produce oxidative secondary products that can be harmful to human health. The ability to accurately predict the shelf life of consumables is very important in the food industry, because a decrease in the shelf life has direct impacts on food quality and human health. Changes in food quality (shelf life) are traditionally predicted using conventional methods, which require extensive experimentation with long cycles and are costly, time-consuming, and labour-intensive [8]. Therefore, to accelerate the experimental process and reduce the experimental time, the accelerated storage technique in combination with the commonly used Arrhenius model has been applied to evaluate the changes in food nutritional quality and predict the shelf life of products during storage [9], previous study demonstrated that the shelf life of dried food products are often evaluated using PV as the indicator [9]. Therefore, it is suggested that more attention should be paid to the lipid oxidation and quality control of the shelf life of green coffee beans.

The accelerated storage technique has been widely used to reduce the experimental time of long-term evaluations and is a very effective tool to study the change characteristics of agricultural products during storage [10]. The Arrhenius equation is the most common model used to evaluate the predictions of quality decline during storage and it is essential to predict the nutritional quality changes that occur during storage. Rendón et al. attributed the observed chemical changes of the sensory characteristics of coffee beans during 15 months of storage, which caused their loss of commercial value, to lipid oxidation [11]. Ribeiro et al. investigated the physical, chemical, and sensory qualities of green coffee beans during storage in different types of packaging [12]. Edible biopolymer coating was used as a barrier coating to preserve the water content, colour, and sensory quality, and the results indicated that the use of starch, 3 h of pre-drying, and a single immersion was the best protocol to retain the colour and water contents of coated green coffee beans [13]. To the best of our knowledge, very limited information is available regarding the impact of accelerated storage on the lipids composition of green coffee beans.

The objective of this study was to investigate the changes in the lipids of green coffee beans during accelerated storage. The acid value (AV), iodine value (IV), *p*-anisidine value (P-AV), peroxide value (PV), total oxidation value (TOTOX), thiobarbituric acid reactive substances (TBARS), free fatty acids (FFA), absorbance at 232 (*K_232 nm_*) and 268 (*K_268 nm_*) nm, and fatty acid composition were all determined. With the aid of unsupervised principal component analysis (PCA) and Hierarchical cluster analysis (HCA), the dynamic distribution and difference analysis of green coffee beans were explored using a fused data set as the input variables during accelerated storage. The Arrhenius model was used to construct an oxidation kinetics equation for the prediction of the shelf life of samples. In addition, this study provides valuable reference data for researchers to design accelerated experiments to predict the shelf life of tropical beverage products.

## 2. Results and Discussion

### 2.1. Changes in MC during Accelerated Storage

MC is a key quality parameter and important to determine because it affects the shelf life of green coffee beans. During storage, the changes in the quality of green coffee beans are mostly related to the process of moisture loss because it may affect the chemical composition and sensory attributes [14]. The changes in the MC of coffee beans during accelerated storage at 40 °C, 50 °C, and 60 °C are shown in Figure S1 (Supplementary material). The initial MC of the coffee beans was 7.88 ± 0.17 g/100 g DW. The MC decreased over the 20 days of accelerated storage at 40 °C, 50 °C, and 60 °C to final values of 3.82 ± 0.24, 3.45 ± 0.20, and 1.85 ± 0.16 g/100 g DW, respectively.

### 2.2. Changes in Oxidative Indexes during Accelerated Storage

#### 2.2.1. Acid Value

**Figure 1:**
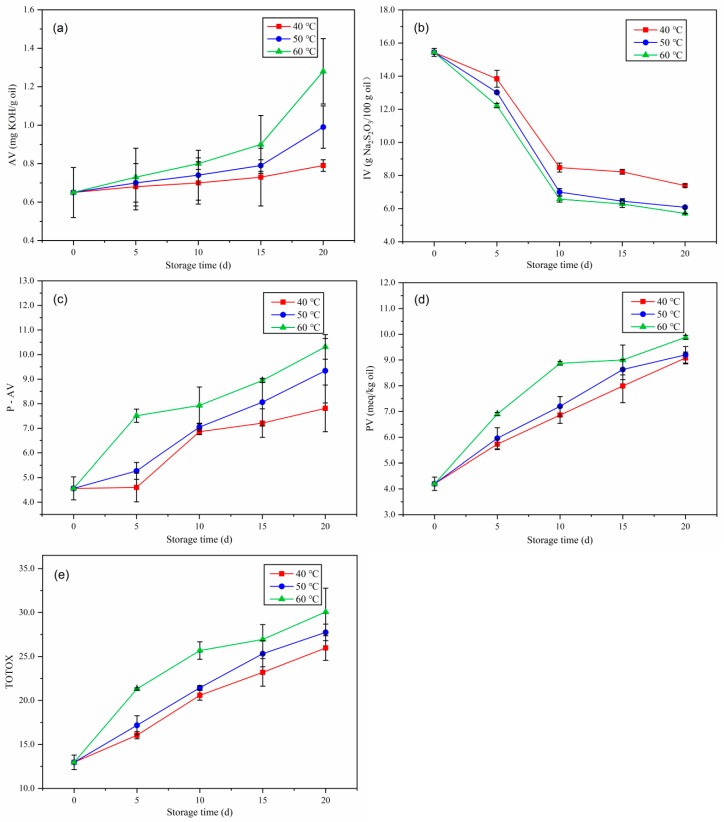
Effects of the different degrees of oxidation of oil on the (**a**) acid value (AV), (**b**) iodine value (IV), (**c**) *p*-anisidine value (P-AV), (**d**) peroxide value (PV), and (**e**) total oxidation value (TOTOX) of green coffee beans during accelerated storage.

The AV is an important index to evaluate the quality of oil since it measures the content of FFAs formed after the hydrolytic degradation of lipid molecules, and can be used to indicate the degree of rancidity in oil hydrolysis [15]. The AV of coffee beans during accelerated storage are shown in Figure 1a. The AV of the coffee oil samples was less than 1.28 mg KOH/g oil in each storage stage, the AV showed a time-dependent increase throughout the 20 days of storage. The AVs of samples at 40 °C, 50 °C, and 60 °C exhibited similar variation trends throughout the accelerated storage period. After accelerated storage for 20 days at 40 °C, 50 °C, and 60 °C, the AVs had increased to 0.79 ± 0.03, 0.99 ± 0.11, and 1.28 ± 0.17 mg KOH/g oil, respectively. Moreover, the change rate of AV from tested samples was faster at the higher storage temperature.

#### 2.2.2. Iodine Value

During storage, the double bonds of USFAs are attacked by free radicals, and thus form conjugated bonds [16]. The IV can be used to determine the degree of unsaturation in the fatty acids, whereby the higher the IV, the fewer conjugated bonds formed. The effect of temperature on the IV of coffee oil during accelerated storage is shown in Figure 1b. Oxidation of the coffee oil increased with the increase in storage time, and the IV of the samples stored at the three temperatures decreased gradually throughout the 20 days storage. This decline was mainly due to the destruction of the fatty acid double bonds as a result of lipid oxidation. On day 20, the IV of coffee oil stored at 40 °C, 50 °C, and 60 °C reached minimums of 7.39 ± 0.09, 6.08 ± 0.06, and 5.71 ± 0.01 g Na_2_SO_3_/100 g oil, respectively. These findings indicated that the higher the storage temperature, the faster the oxidation rate. Similar IV decreasing trends have been reported by Guo et al. in palm oil with rosemary ethanol extract, and Jahurul et al. in mango seed fat and palm oil mid-fraction blends as cocoa butter replacers [17,18].

#### 2.2.3. *p*-Anisidine Value

The P-AV analysis is a good method to evaluate the secondary lipid oxidation of oil. Generally, aldehyde carbonyl bonds are formed during secondary lipid oxidation [17]. Primary oxidation products are colourless and odourless, and the secondary oxidation products have odours. In this study, as expected, there was a significant increase in the P-AV throughout the accelerated storage, as shown in Figure 1c. Lower P-AVs indicated a lower production of rancid oil. The P-AV at the beginning of the storage period was 4.56 ± 0.47. After accelerated storage for 20 days at 40 °C, 50 °C, and 60 °C, the P-AVs reached 7.81 ± 0.95, 9.34 ± 1.31, and 10.31 ± 0.50, respectively. These findings indicated that a higher amount of secondary ethanol extract was generated at the higher storage temperature. Lipid oxidation is one of the major causes of quality deterioration of many foods, leading to the rancid flavour of oil. These findings were consistent with those reported by Wang et al., who demonstrated an increase in the P-AVs of samples with the increase in storage period in sunflower oil flavoured with essential oil from *Coriandrum sativum* L. during accelerated storage [10].

#### 2.2.4. Peroxide Value

An abundance of primary products, peroxides and hydroperoxides, were formed in oils via autoxidation during the oxidation process [19]. The PV can be used as an oxidative index for the early stages of lipid oxidation, and a higher oxidative stability is usually accompanied by a slower increase in the PV. The effect of storage temperature on the evolution of PVs throughout the 20-day storage period of green coffee beans is shown in Figure 1d. The PV of the samples increased almost linearly with the increase in storage time. The initial PV of the green coffee beans was 4.20 ± 0.26 meq/kg oil. The PVs were found to differ significantly with relation to the storage time. After 20 days of accelerated storage at 40 °C, 50 °C, and 60 °C, the PVs increased to 9.08 ± 0.23, 9.20 ± 0.32, and 9.88 ± 0.08 meq/kg oil, respectively. The PVs increased with the increase in hydrogen peroxide during storage, and the high PVs of coffee oil samples indicated their low oxidative rancidity during storage. Our findings were consistent with those of Chong et al., who reported a remarkable rise in the PV of sunflower oil with mangosteen peel extract after 18 days of accelerated storage [20].

#### 2.2.5. Total Oxidation

The TOTOX of coffee oil was determined using the PV and P-AV values. It comprises the information for primary and second oxidation analyses, indicates the overall oxidation state of the assessed oils [21], and thus enables the information regarding the oxidative deterioration progress to be traced more clearly. Figure 1e depicts the changes in the TOTOX value of the coffee oil throughout accelerated storage. The change trends of TOTOX were seen to be similar to those of the PV values, whereby the TOTOX values of coffee oil increased regularly over the storage period. The initial TOTOX was 12.97 ± 0.82. After 20 days of storage at 40 °C, 50 °C, and 60 °C, the TOTOX had increased to ~25.97 ± 1.40, 27.75 ± 0.94, and 30.07 ± 2.71, respectively. These findings demonstrated that the experimental temperatures were effective in increasing the formation of secondary oxidation products, and thus reducing the oxidative stability. Furthermore, the lower the TOTOX value, the better the quality of oil, thus samples treated after accelerated conditioning at 40 °C would be of higher quality. Lower values of this index were also found in horchata oil and tiger nut oil [22,23].

### 2.3. Changes in Thiobarbituric Acid and FFA Contents during Accelerated Storage

#### 2.3.1. Thiobarbituric Acid (TBARS) Analysis

**Figure 2:**
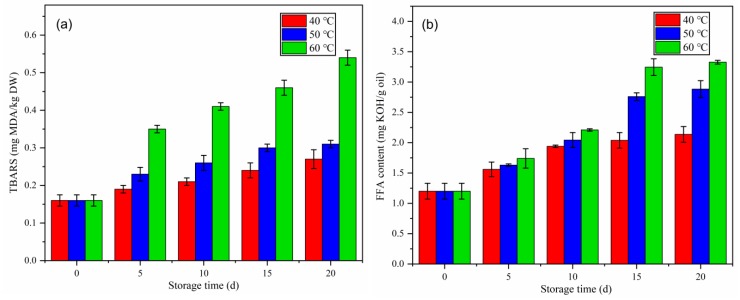
Changes in the thiobarbituric acid reactive substances (TBARS) (**a**) and free fatty acid (FFA) content (**b**) in green coffee beans during accelerated storage.

TBARS is defined by the content of malondialdehyde (MDA) (mg) present in 1 kg of sample and TBARS analysis is the most widely used method to determine the secondary oxidation products [24,25]. The primary oxidation products, i.e., lipid hydroperoxides that reacted with oxygen to form MDA, generate the off-flavour of oil. The TBARS values of coffee beans during accelerated storage are shown in Figure 2a. The increase in the TBARS values of the samples was slowest in those stored at 40 °C, followed by those stored at 50 °C, and fastest in those stored at 60 °C. The initial TBARS value was 0.16 mg MDA/kg DW. After 20 days of storage at 40 °C, 50 °C, and 60 °C, the TBARS values of the coffee bean samples were 0.27, 0.31, and 0.54 mg MDA/kg DW. Food products with TBARS values <0.576 mg MDA/kg DW are considered to be fresh, those with TBARS values of 0.65–1.44 mg MDA/kg DW are considered rancid but are still acceptable, and those with TBARS values >1.5 mg MDA/kg DW are considered rancid and unacceptable for consumption [26]. Similar results were reported for other products, for example, Xie et al. reported the TBARS values of two clam tissues during accelerated storage for 20 days at 50 °C and 60 °C and showed that higher values were observed at higher storage temperatures [8]. It is noteworthy that the findings of the TBARS analysis differed from those of the P-AV test, which measured the content of aldehydes; i.e., the TBARS analysis is more sensitive and may be affected by ketones, esters, pyridines, and other compounds.

#### 2.3.2. Free Fatty Acid Analysis

The FFA content can be used as a measure of the hydrolytic rancidity that occurs either by enzymatic or spontaneous hydrolysis of triglycerides, and FFA is usually used as an indicator of fat hydrolysis [27]. The initial FFA content was 1.20 ± 0.13 mg KOH/g oil. The FFA content of all samples increased over the 20 days of storage and were significantly different among the different temperature treatments. The total increase in FFAs was highest in samples stored at 60 °C, lower in samples stored at 50 °C, and lowest in samples stored at 40 °C throughout the 20 days of accelerated storage (Figure 2b). These findings may be due to the hydrolytic activity of lipophilic enzymes being more effective at the higher temperatures. After 20 days of storage at 40 °C, 50 °C, and 60 °C, the FFA content of coffee samples had increased to 2.14 ± 0.12, 2.88 ± 0.14, and 3.33 ± 0.03 mg KOH/g oil, respectively. In accordance with our findings, Rendón et al. reported that the FFA content varied throughout storage and that 3.84 mg KOH/g oil (CD) and 3.67 mg KOH/g oil (CN) was retained at the end of 15 months of storage [11]. In general, FFA is a more useful indicator of rancidity development and possibly comprised quality in green coffee beans placed in atmospheric moisture.

### 2.4. Changes in K232 nm and K268 nm during Accelerated Storage

In general, hydrogen peroxide is formed during the early stages of oxidation and is absorbed at 232 nm. Absorbance at 232 nm can, therefore, be used to measure the degree of primary oxidation, and *K_232 nm_* is a measure of conjugated dienes [28]. Figure S2a (Supplementary material) indicated that the *K_232 nm_* value increased with the increase in storage time, which mainly resulted from the increasing trend of dienes throughout the 20 days of storage. After 20 days of storage at 40 °C, 50 °C, and 60 °C, the *K_232 nm_* values increased to 0.99 ± 0.01, 1.40 ± 0.02, and 1.57 ± 0.01, respectively. 

Absorbance at 268 nm is used to detect the secondary oxidation of oil, which is not completely similar to the analysis of anisidine, because the ketones are not detected in anisidine determination. At the same time, more and more ketones were generated with the extension of storage time. This difference arose from smaller increases in the content of conjugated trienes at the late stages of lipid oxidation. The influence of accelerate storage on *K_268 nm_* values are shown in Figure S2b (Supplementary material). After 20 days of storage at 40 °C, 50 °C, and 60 °C, the *K_268 nm_* values clearly increased to 0.98 ± 0.01, 1.20 ± 0.02, and 1.32 ± 0.02, respectively. In the present study, the *K_232 nm_* and *K_268 nm_* values showed a similar change patterns to that of the FFA content, but slightly different from that of the PVs. This difference is ascribed to the conjugated dienes that were mainly generated from polyunsaturated fatty acids, since fatty acid hydroperoxides determined by the PVs were developed from mono- and polyunsaturated fatty acids. Similarly, Franklin et al. showed that the higher the polyunsaturated acids content, the more conjugated dienes and trienes were formed during accelerated storage in roasted almonds [29].

### 2.5. The Effects of Accelerated Storage on the Fatty Acid Content

The fatty acid compositions of green coffee beans at different storage temperatures are shown in Table 1. The fatty acids detected in this study were: pentadecanoic acid (C15:0), palmitic acid (C16:0), margaric (C17:0), stearic acid (C18:0), oleic acid (C18:1), linoleic acid (C18:2), ɑ-linolenic acid (C18:3), arachidic acid (C20:0), eicosenoic acid (C20:1), eicosadienoic acid (C20:2), and heneicosanoic acid (C21:0). The major fatty acids present in the samples were palmitic acid (107.95 ± 0.01 mg/g oil), oleic acid (109.94 ± 1.47 mg/g oil), stearic acid (354.45 ± 1.77 mg/g oil), and linoleic acid (2.26 ± 0.01 mg/g oil), which was consistent with the findings of our previous studies [5,30]. The accelerated storage led to a decrease in the content of saturated fatty acids (SFAs) and USFAs, and a higher reduction was observed at the higher storage temperatures. This decrease may be due to the effects of hydrolysis and oxidation throughout the storage time of FFAs in the coffee beans. However, the reverse was true for the SFAs. After 20 days of accelerated storage at 40 °C, 50 °C, and 60 °C, the SFA content of samples increased to 3.23 ± 1.26, 7.76 ± 1.17, and 14.21 ± 3.14 mg/g oil, respectively, while the USFA contents of samples were 100.05 ± 3.73, 135.64 ± 14.13, and 97.28 ± 13.50 mg/g oil, respectively. These differences possibly resulted from the high proportion of SFAs altering the oxidation stability. Especially, a high proportion of polyunsaturated fatty acids has a negative effect on the quality of the final product, because these compounds are easily broken down via the double bonds, which stimulates the formation of odour compounds.

### 2.6. Principal Component Analysis

**Figure 3:**
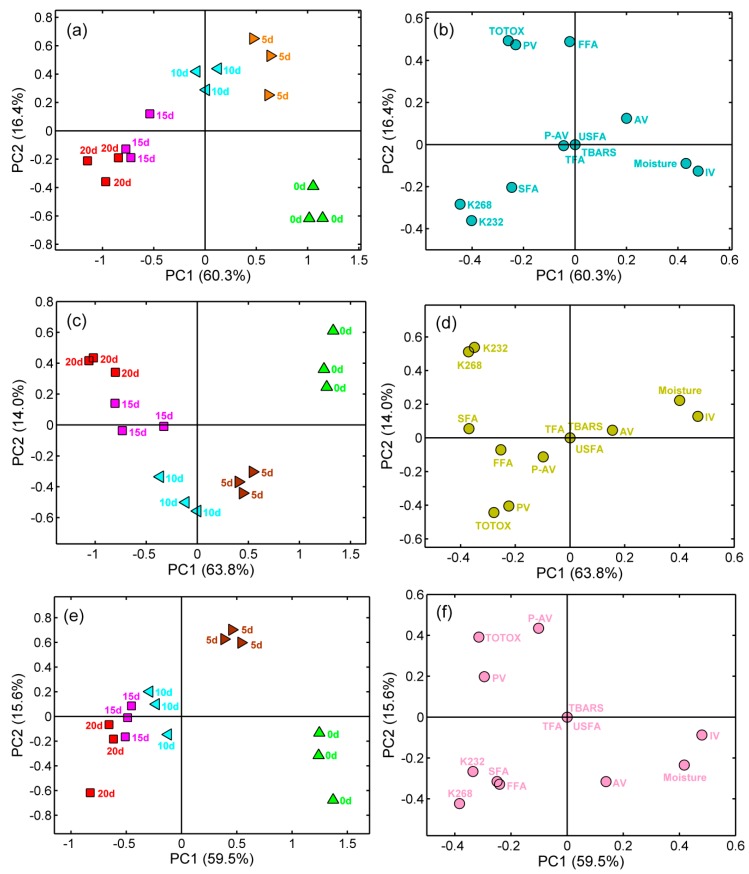
Component (PC) analysis of all tested samples showing a correlation of measured quality parameters during accelerated storage at 40 °C, PC1-PC2 score plot (**a**) and loading plot (**b**); 50 °C, PC1-PC2 score plot (**c**) and loading plot (**d**); 60 °C, PC1-PC2 score plot (**e**) and loading plot (**f**).

PCA is a mathematical transformation used to extract the variance in a data matrix, which is then projected in a reduced-dimension plot to highlight the similarities and differences [30]. In the present study, the original data was scaled in the range of –1 to 1 before PCA to eliminate the effect of index dimension. The PCA was performed with the fused dataset (MC, AV, IV, P-AV, PV, TOTOX, TBARS, FFA, *K*_232 nm_, *K*_268 nm_, SFA, USAF, and total fatty acids (TFA) of green coffee beans stored at 40 °C, 50 °C, and 60 °C. The first two principal components (PCs) explained ~76.7%, ~77.8%, and ~75.1% of the total variance of the data when the fused datasets were applied as input variables. When all samples were projected onto the two-dimensional space, the samples were differentiated in a storage time-dependent pattern. Samples stored at 0 d, 5 d, and 10 d were completely discriminated, but overlaps existed between the coffee samples stored at 15 d and 20 d (Figure 3a–f). Furthermore, IV and MC had positive loadings on PC1, while *K*_232 nm_ and *K*_268 nm_ had negative loadings on PC1. PC2 was mainly positive correlated with TOTOX and PV for samples stored at 40 °C and 60 °C, whereas negatively correlated with TOTOX and PV for samples stored at 50 °C. Therefore, the PCA results demonstrated that the fused dataset of green coffee beans can be clustered based on storage time, although with some minor overlaps. Moreover, some samples from the different storage times were grouped closely, which may have been the result of similar lipid profiles and oxidative indexes. The application and potential of hierarchical cluster analysis to overview the spatial distribution and relationship between oxidative indexes and samples should be further evaluated.

### 2.7. Hierarchical Clustering Analysis

**Figure 4:**
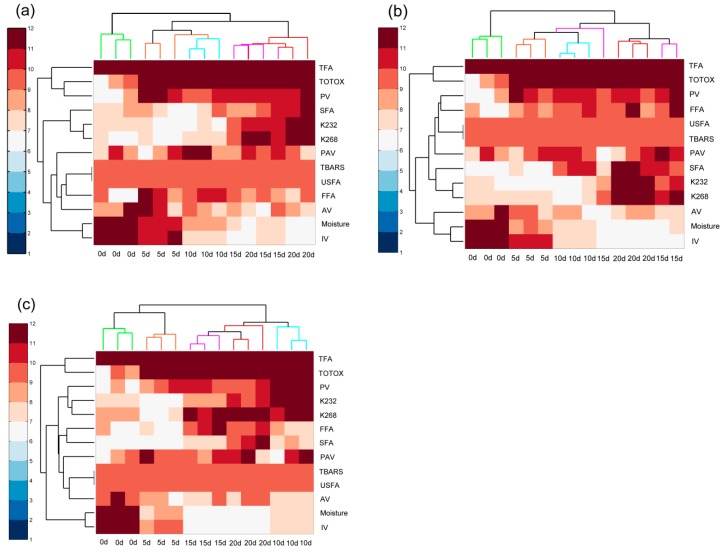
HCA of green coffee beans during accelerated storage based on the fused dataset (**a**: 40 °C; **b**: 50 °C; **c**: 60 °C). TFA, trans fatty acid; TOTOX, total oxidation value; PV, peroxide value; K232, the absorbance at 232 nm; K268, the absorbance at 268 nm; FFA, free fatty acids; SFA, saturated fatty acids; PAV, *p*-anisidine value; TBARS, thiobarbituric acid reactive substances; USFA, unsaturated fatty acids; AV, acid value; Moisture, moisture content; IV, iodine value.

HCA was performed using squared between-groups linkages and the Euclidean distance was applied to measure the homogeneity of groups among green coffee samples based on the different storage times. A heat map was made using the lipid profile fused dataset. The clustering results were similar to those in the PCA biplot depicted in Figure 3a–f. As shown in Figure 4a, all samples were grouped into three clusters, which indicated that there were noteworthy differences among coffee samples at different times of the accelerated storage at 40 °C. The first cluster was the fresh green coffee beans, while the second cluster consisted of samples stored for 5 d and 10 d, and samples stored for 15 d and 20 d were in the third cluster.

Similarly, the green coffee beans stored under accelerated storage at 50 °C and 60 °C also formed three clusters. As shown in Figure 4b, three distinguishable clusters were observed in the heat map, one sample from 15 d storage was located in the cluster of 5 d and 10 d group. As shown in Figure 4c, green coffee samples were divided into four major clusters, expectedly: fresh samples, samples stored for 5 d and 20 d, samples stored for 10 d, and samples stored for 15 d. Furthermore, the fresh coffee samples were more characterised by high MCs and IVs, and samples stored for 5 d and 10 d were characterized by high AVs and P-AVs. Moreover, the samples stored for 15 d were particularly characterised by the PV and SFA content, and the samples stored for 20 d were indicated by the *K*_232 nm_ and *K*_268 nm_ indexes.

### 2.8. Shelf Life Prediction

Zero order and first order kinetic models were used to construct the food quality reaction kinetic models. The Arrhenius formula showed that ln(k) had a linear relationship with 1/T, and the first-order model fitted the changes in PV better than the zero-order model, based on the value of *R^2^* shown in Table S1 (Supplementary Material). The Arrhenius equation was: In(k) = −2267.5/T + 4.0191, and the predicted shelf life for green coffee bean stored at 40 °C, 50 °C, and 60 °C were 57.39 days, 44.44 days, and 23.12 days, respectively. This model can be used to evaluate the quality of coffee beans after storage at a certain temperature and storage time, and it can also be utilized to obtain the actual storage time corresponding to a certain quality value [31]. Results indicated that the higher the temperature, the shorter the induction period. This model offers a faster and more accurate way to predict the shelf life of green coffee beans stored at different temperatures.

## 3. Materials and Methods

### 3.1. Chemicals and Reagents

Petroleum ether, phenolphthalein, potassium hydroxide, iodine, sodium thiosulphate, 2,2,4-trimethylpentane, *p*-anisidine, acetic acid, ethylenediamine tetraacetic acid disodium (EDTA), trichloroacetic acid, 4,6-dihydroxy-2-mercaptopyrimidine (TBA), and dichloromethane were purchased from Aladdin Reagent Co., Ltd. (Shanghai, China). HPLC grade methanol was obtained from Merck (Darmstadt, Germany). Fatty acid methyl esters (FAMEs) and *n*-heptane were supplied by Sigma-Aldrich Chem. Co. (St. Louis, MO, USA). Besides, the reagents and chemical materials used in this study were of analytical or HPLC grade and provided by chemical suppliers from China.

### 3.2. Samples Preparation and Accelerated Storage Treatment

Samples were prepared following the methods of Franklin et al. with minor revisions [29]. Coffee beans were harvested in 2018/2019 at the experimental base of the Spice and Beverage Research Institute of the Chinese Academy of Tropical Agricultural Sciences (Wanning, China). Fresh, non-perishable coffee fruits were selected for this study. The harvested raw coffee beans were obtained by mechanical dehulling, degumming, peeling, and drying. Before accelerated storage tests, the dried green coffee beans were divided into three batches which were each further split into 250 samples that were stored in open brown paper bags. Samples were then transferred into three hot-air convection incubators (DNP-9162BS-Ⅲ, Xinmiao Instrument, Shanghai, China) set at 40 °C, 50 °C, and 60 °C and incubated for 20 days to mimic accelerated storage conditions. Nine subsamples of each coffee sample were randomly withdrawn from the incubator every 5 days and repackaged into polyethylene vacuum sealed packages, which were stored at −80 °C in an ultra-low temperature freezer (DW-86L728J, Haier Inc., Qingdao, China) until analysis. Coffee samples (~80 g) were thawed and ground for 30 s using a grinder (RT-2, 650 W, Wuyi Medicine Machine, Wuyi, China). Ground samples were sieved through a 40-mesh sieve (GB/T 6003, Shangyu Shenchao Instrument, Shaoxing, China) and then stored in vacuum-sealed plastic bags at 4 °C until further analysis, within 1 week.

### 3.3. Determination of Moisture Content (MC)

The MC of the samples was determined according to the National Standard of China [32]. In brief, 2 g of raw coffee powder was dried at 105 °C until a constant weight was reached, and the MC was estimated as the difference in mass and expressed on a dry weight basis. The MC was calculated as follows:

MC = (*m_i_* − *m_d_*)/*m_i_* × 100% (1)

where *m_i_* is the initial weight of the coffee samples before drying, and *m_d_* is the weight of the coffee samples after drying.

### 3.4. Coffee Oil Extraction

In brief, 50 g of coffee powder was mixed with 400 mL petroleum ether in a conical flask at room temperature (25 °C) in an ultra-sonic bath (SONIC-T20, Guangdong Gute Inc., Shenzhen, Guangdong, China) for 30 min. The sample was then centrifuged at 3000 rpm for 10 min. The organic phase was collected, and the solvent was evaporated using an R-215 vacuum rotatory evaporator (Buchi Instrument, Flawil, Switzerland). The extraction oil was collected and transferred to a 50 mL amber borosilicate glass sample vial with a polytetrafluoroethylene-faced rubber lined cap and stored at –80 °C until further analysis, within 1 week.

### 3.5. Determination of Oxidative Indexes

#### 3.5.1. Acid Value (AV)

The AV was measured according to the National Standard of China [33]. In brief, a 1 g of oil sample was added to ether-isopropyl alcohol (1:1, *v*/*v*) and the mixture was titrated against KOH (0.1 M) using phenolphthalein solution (10 g/L) as an indicator, i.e., when the pink colour of the phenolphthalein persisted for at least 30 s. AV was expressed as the milligrams of potassium hydroxide required to neutralize the FFAs present in 1 g of the oil sample (mg KOH/g oil).

#### 3.5.2. Iodine Value (IV)

The IV was measured according to the National Standard of China [34]. A 0.4 g sample of coffee oil was mixed with 25 mL of Wijs reagent (1:1, *v*/*v*) and 20 mL of hexamethyleneacetic acid (1:1, *v*/*v*) in a 500 mL iodine determination flask and reacted for 1 h in the dark. Thereafter, 20 mL of potassium iodide, 150 mL of water, and a few drops of starch solution were added. The mixture was titrated against Na_2_S_2_O_3_ (0.1 M) until the blue colour disappeared and the results were expressed as g Na_2_S_2_O_3_/100 g oil.

#### 3.5.3. *p*-Anisidine Value (P-AV)

The P-AV analyses were conducted according to the National Standard of China [35]. Briefly, a 0.6 g sample of coffee oil was mixed with 80 mL of 2,2,4-trimethylpentane. A 5 mL sample of the resulting solution and 1 mL of *p*-anisidine solution were thoroughly mixed and left to react for 10 min in the dark. The absorbance was recorded at 350 nm using a Specord 250 Plus spectrophotometer (Analytik Jena, Jena, Germany).

#### 3.5.4. Peroxide Value (PV)

The PV was measured was according to the National Standard of China [36]. Briefly, a 1 g sample of coffee oil was mixed with 30 mL chloroform-acetic acid (2:3, *v*/*v*). Saturated potassium iodide (0.5 mL) was added and the mixture allowed to react for 3 min in the dark. Thereafter, 50 mL of water and 0.5 mL 1% starch solution were added. Finally, the mixture was titrated with sodium thiosulphate solution (0.01 M) until the blue colour disappeared. Results were expressed as peroxide milliequivalent per kg oil (meq/kg oil).

#### 3.5.5. Total Oxidation Value (TOTOX)

The overall oxidative state of the coffee oil can be evaluated by calculating the TOTOX, that is, both the primary and secondary oxidation products, the TOTOX can provide a better description of the overall quality of the oil. The TOTOX was calculated using the following equation as described by Chew et al. [37]:

TOTOX = P-AV + 2PV(2)

where P-AV is the *p*-anisidine value, PV is the peroxide value.

### 3.6. Measurement of Thiobarbituric Acid Reactive Substances (TBARS)

The TBARS was determined according to the National Standard of China [38]. A 4 g sample of coffee oil was added to 50 mL of a mixed trichloroacetic acid solution containing 0.5 g of ethylenediamine tetraacetic acid disodium (EDTA). The reaction mixture was heated for 30 min in a water bath at 50 °C and cooled in an ice bath. A 5 mL sample of the solution was mixed with 5 mL of mixed trichloroacetic acid solution and 4,6-Dihydroxy-2-mercaptopyrimidine (TBA) in a water bath at 90 °C and then cooled in an ice bath. The absorbance was recorded at 532 nm using a Specord 250 Plus spectrophotometer (Analytik Jena) and the results were expressed as mg MDA/kg DW. 

### 3.7. Measurement of Free Fatty Acids (FFA)

A 1 g sample of coffee oil was dissolved in 20 mL of a mixed dichloromethane/ethanol (1/1, *v*/*v*) solution. Phenolphthalein was used as the indicator. The resulting organic solution was stirred with a magnetic stir bar and titrated with 0.1 M potassium hydroxide solution until the mixture solution turned pink. The FFA content was expressed as mg KOH/g oil [39].

### 3.8. Measuring Absorbance at 232 (K232 nm) and 268 nm (K268 nm)

Absorbance was measured at 232 nm and 268 nm according to the National Standard of China [40]. In brief, 0.1 g of green coffee oil was place into a 25 mL volumetric flask and made up to volume with isooctane. A 1 mL sample of the solution was then added to 4 mL isooctane and the absorbance measured at 232 nm and 268 nm to determine the conjugated dienes and trines, and the oxidation product during the accelerated storage. Isooctane was used as the blank.

### 3.9. Fatty Acids Analysis by GC-MS

The fatty acid composition was analysed according to the National Standard of China [41]. To quantify the FAMEs, samples were transesterified using sodium hydroxide in methanol (2.0%). The FAMEs obtained were separated with n-heptane, dehydrated with sodium sulphate, and transferred to a vial for qualitative and quantitative analysis. Samples were analysed using an Agilent 7890A gas chromatograph coupled to a 5975C mass spectrometer (Agilent Technologies, Cheshire, UK), equipped with a DB-WAX column (30 m × 0.25 mm I.D. × 0.25 µm df). The oven temperature program was: initial temperature of 130 °C, held for 1 min, increased to 180 °C at 5 °C/min, then increased to 220 °C at 1 °C/min, and held for 10 min. The injector, MS transfer line, and ion source temperatures were all 250 °C. Helium was used as the carrier gas at a flow rate of 1 mL/min and the injector volume was 2 µL. The chromatographic peak area of each fatty acid was quantified with reference to the internal standard undecanoic acid methyl ester and results were expressed as mg/g oil.

### 3.10. Oxidation Kinetics and Shelf Life Prediction

The shelf life prediction of green coffee oil was preformed refers to the method described by Xie et al. [8] with minor modification. The PV of green coffee oil with different storage time at 40 °C, 50 °C, and 60 °C were used to fit the following kinetics models: 

Zero-order model:

PV = *k*_0_*t* + PV_0_ (3)

First-order model:

Ln(PV) = *kt* + ln(PV_0_) (4)

Arrhenius equation:

Ln(*k*) = −*E*_A_/*RT* + ln(*k_0_*) (5)

Shelf life prediction:

SL = [ln(PV_lim_) − ln(PV_0_)]/[*k*_0_ e^(−*EA/RT*)^] (6)

where *k*_0_ and *k* are the reaction rate constants, *PV* and *PV*_0_ are the *PVs* at storage time *t_h_* and the initial value, *k*_0_ is a pre-exponential factor, *E_A_* is the activation energy (J mol^−1^), *T* is the absolute temperature, and R is the molar gas constant (8.3144 J K^−2^ mol^−1^).

### 3.11. Statistical Analysis

All experiments were conducted in triplicate and the results were expressed as means ± standard deviations (SDs). The significance of differences was tested by one-way analysis of variance (ANOVA) with a Tukey post-hoc test (*p* < 0.05) using SPSS 22.0 software (SPSS, Chicago, IL, USA). Shapiro-Wilk and Levene’s tests were used to test the normality of the data. The PCA and hierarchical clustering analysis (HCA) were performed using MATLAB R2010a software (Mathworks, Natick, MA, USA). Before importing the data into MATLAB for the PCA and HCA, all data were auto-scaled to eliminate dimensional effects.

## 4. Conclusions

The MC, oxidative indexes, and fatty acid composition changes of green coffee beans during accelerated storage for 20 days at different temperatures were examined. Lipid oxidation occurred in coffee samples during accelerated storage. The AV, P-AV, PV, TOTOX, TBARS, FFA, *K*_232 nm_, *K*_268 nm_, and SFA content increased, while the MC, IV, and USFA content decreased throughout storage. Multivariate statistical techniques (PCA and HCA) were used to characterize the samples based on the storage duration, and the results demonstrated that most of the samples showed clustering according to their unique attributes. The PCA two-dimensional spatial distribution was consistent with that of the heat map analysis. Moreover, the accelerated storage experiment in combination with Arrhenius equation modelling was appropriate for the shelf life prediction of green coffee beans. All indexes demonstrated that storage resulted in the deterioration in the quality of green coffee beans, however, more in-depth studies are required to confirm this notion.

## Figures and Tables

**Table 1:** Changes in fatty acid content of green coffee beans during accelerated storage at various temperatures.

Fatty Acids (mg/g oil)	Days	40 °C	50 °C	60 °C
C15:0	0	0.25 ± 0.01b	0.25 ± 0.01a	0.25 ± 0.01c
	5	0.26 ± 0.35b	0.23 ± 0.03a	0.37 ± 0.15c
	10	0.30 ± 0.09b	0.26 ± 0.10a	1.57 ± 0.16b
	15	1.16 ± 0.49a	0.34 ± 0.02a	1.66 ± 0.01b
	20	1.06 ± 0.54a	0.26 ± 0.06a	4.89 ± 1.83a
C16:0	0	109.75 ± 0.01a	109.75 ± 0.01a	109.75 ± 0.01a
	5	73.53 ± 1.99b	79.96 ± 0.01b	55.85 ±0.01b
	10	70.80 ± 0.40b	75.22 ± 6.00bc	51.55 ± 1.86b
	15	60.59 ± 2.10c	67.96 ± 3.55cd	42.21 ± 0.01c
	20	38.46 ± 2.56d	61.48 ± 4.97d	45.48 ± 4.28c
C17:0	0	0.13 ± 0.03b	0.13 ± 0.03c	0.13 ± 0.03b
	5	0.17 ± 0.01b	0.27 ± 0.04c	0.12 ± 0.03b
	10	0.19 ± 0.06b	0.29 ± 0.07c	0.20 ± 0.12b
	15	0.35 ± 0.21b	1.13 ± 0.45b	0.39 ± 0.06b
	20	1.15 ± 0.44a	1.96 ± 0.38a	0.88 ± 0.35a
C18:0	0	109.94 ± 1.47a	109.94 ± 1.47a	109.94 ± 1.47a
	5	65.97 ± 0.14b	73.13 ± 5.37b	42.51 ± 2.39b
	10	32.60 ± 0.01c	68.62 ± 5.61bc	42.27 ± 0.11b
	15	32.06 ± 0.22c	60.97 ± 1.06c	31.30 ± 1.53c
	20	25.16 ± 2.58d	45.84 ± 2.55d	21.12 ± 5.95d
C18:1	0	2.26 ± 0.01a	2.26 ± 0.01a	2.26 ± 0.01a
	5	1.12 ± 0.24b	0.51 ± 0.03b	0.28 ± 0.04b
	10	1.02 ± 0.18b	0.28 ± 0.01c	0.25 ± 0.17b
	15	0.38 ± 0.10c	0.25 ± 0.08c	0.13 ± 0.02b
	20	0.14 ± 0.03c	0.23 ± 0.02c	0.12 ± 0.01b
C18:2	0	354.45 ± 1.77a	354.45 ± 1.77a	354.45 ± 1.77a
	5	69.21 ± 9.72b	57.75 ± 1.51b	50.35 ± 9.99bc
	10	64.00 ± 0.10b	45.84 ± 2.55c	46.64 ± 1.10c
	15	50.18 ± 1.10c	50.32 ± 1.64bc	62.78 ± 1.00b
	20	35.53 ± 1.00d	25.64 ± 7.00d	28.19 ± 6.30d
C18:3	0	2.00 ± 0.60a	2.00 ± 0.60ab	2.00 ± 0.60ab
	5	2.40 ± 0.02a	2.50 ± 0.36a	1.27 ± 0.33b
	10	2.17 ± 0.02a	0.83 ± 0.07c	2.68 ± 0.40a
	15	0.49 ± 0.13b	1.65 ± 0.09b	1.44 ± 0.70b
	20	0.26 ± 0.08b	1.54 ± 0.17b	1.41 ± 0.11b
C20:0	0	0.31 ± 0.03c	0.31 ± 0.03c	0.31 ± 0.03c
	5	0.25 ± 0.03c	0.54 ± 0.01c	0.69 ± 0.17c
	10	0.45 ± 0.01bc	1.70 ± 0.02b	0.70 ± 0.12c
	15	0.69 ± 0.01ab	1.96 ± 0.84b	2.62 ± 0.18b
	20	0.72 ± 0.17a	4.79 ± 0.71a	7.24 ± 0.40a
C20:1	0	2.36 ± 0.84a	2.36 ± 0.84a	2.36 ± 0.84a
	5	1.16 ± 0.07b	1.10 ± 0.16b	0.47 ± 0.01c
	10	0.95 ± 0.01c	1.17 ± 1.02b	0.07 ± 0.01d
	15	0.56 ± 0.03d	0.56 ± 0.25c	0.43 ± 0.01c
	20	0.10 ± 0.02e	0.75 ± 0.02c	0.83 ± 0.01b
C20:2	0	0.48 ± 0.01a	0.48 ± 0.01a	0.48 ± 0.01b
	5	0.10 ± 0.03b	0.47 ± 0.11a	0.14 ± 0.00d
	10	0.45 ± 0.22a	0.33 ± 0.26b	0.79 ± 0.46a
	15	0.10 ± 0.09b	0.32 ± 0.30b	0.31 ± 0.01c
	20	0.40 ± 0.12a	0.16 ± 0.01c	0.12 ± 0.01d
C21:0	0	0.25 ± 0.01a	0.25 ± 0.01c	0.25 ± 0.01b
	5	0.28 ± 0.03a	0.23 ± 0.05c	0.20 ± 0.02b
	10	0.25 ± 0.01a	0.24 ± 0.01c	0.26 ± 0.17b
	15	0.30 ± 0.01a	0.91 ± 0.47a	1.00 ± 0.56a
	20	0.33 ± 0.11a	0.75 ± 0.02b	1.20 ± 0.56a
SFA	0	0.94 ± 0.13cd	0.94 ± 0.13e	0.94 ± 0.13d
	5	0.82 ± 0.41d	1.27 ± 0.12d	1.38 ± 0.37c
	10	1.19 ± 0.75c	4.98 ± 0.23b	4.65 ± 0.63b
	15	2.50 ± 0.71b	4.34 ± 1.78c	4.52 ± 0.79b
	20	3.26 ± 1.26a	7.76 ± 1.17a	14.21 ± 3.14a
USFA	0	654.01 ± 17.00a	654.01 ± 17.00a	654.01 ± 17.00a
	5	213.39 ± 12.49b	215.42 ± 8.68b	150.87 ± 12.45b
	10	172.09 ± 3.60c	192.26 ± 14.53c	144.25 ± 3.86c
	15	144.36 ± 0.63d	182.06 ± 13.31d	138.59 ± 7.48d
	20	100.05 ± 3.73e	135.64 ± 14.13e	97.28 ± 13.50e
TFA	0	654.95 ± 17.13a	654.95 ± 17.13a	654.95 ± 17.13a
	5	214.21 ± 13.10b	216.69 ± 8.80b	152.25 ± 12.82b
	10	173.28 ± 3.77c	197.24 ± 14.76c	148.90 ± 4.49b
	15	146.86 ± 1.34d	186.40 ± 15.09d	143.11 ± 8.27c
	20	103.31 ± 4.99e	143.40 ± 15.30e	111.49 ± 16.64d

Data represent means ± standard errors. SFA, saturated fatty acids; USFA, unsaturated fatty acids; TFA, total fatty acids. Means within a column with the same lowercase letters are not significantly different (*p* < 0.05).

## Supplementary Materials

The following are available online, Figure S1: The effect of temperature and time on the moisture content of green coffee bean during accelerated storage, Figure S2: Changes in absorbance of green coffee beans during accelerated storage at 232 nm (**a**) and 268 nm (**b**); Table S1: The changes in the peroxide values (PVs) at various temperatures during accelerated storage were determined by fitting the data to kinetic models, and shelf life predictions were achieved using the Arrhenius equation.
