# Supplementary material for: Characterization of the Lipid Oxidation Process of Robusta Green Coffee Beans and Shelf Life Prediction during Accelerated Storage
Source: Molecules. 2020 Mar 5;25(5):1157. doi: 10.3390/molecules25051157 (PMC7179143; doi:10.3390/molecules25051157)
Supplement: Supplementary file 1 [file molecules-25-01157-s001.pdf]

Supplementary material

Figure S1. The effect of temperature and time on the moisture content of green coffee bean during accelerated storage.

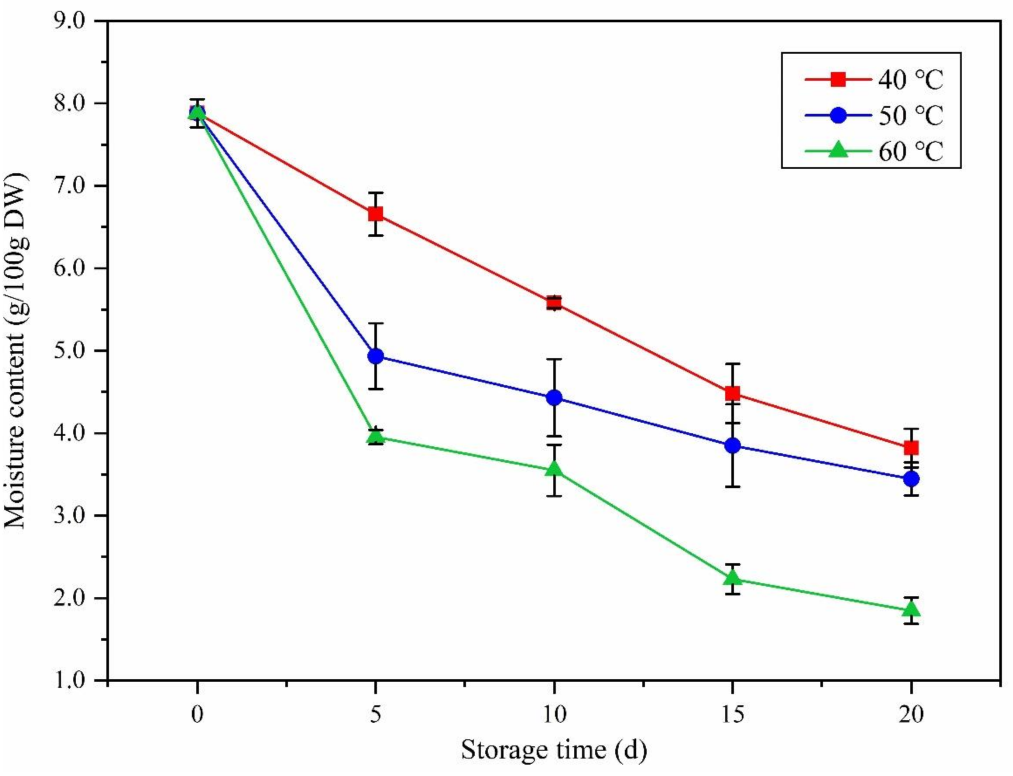

6

Figure S2. Changes in absorbance of green coffee beans during accelerated storage at 232 nm (**a**) and 268 nm (**b**).

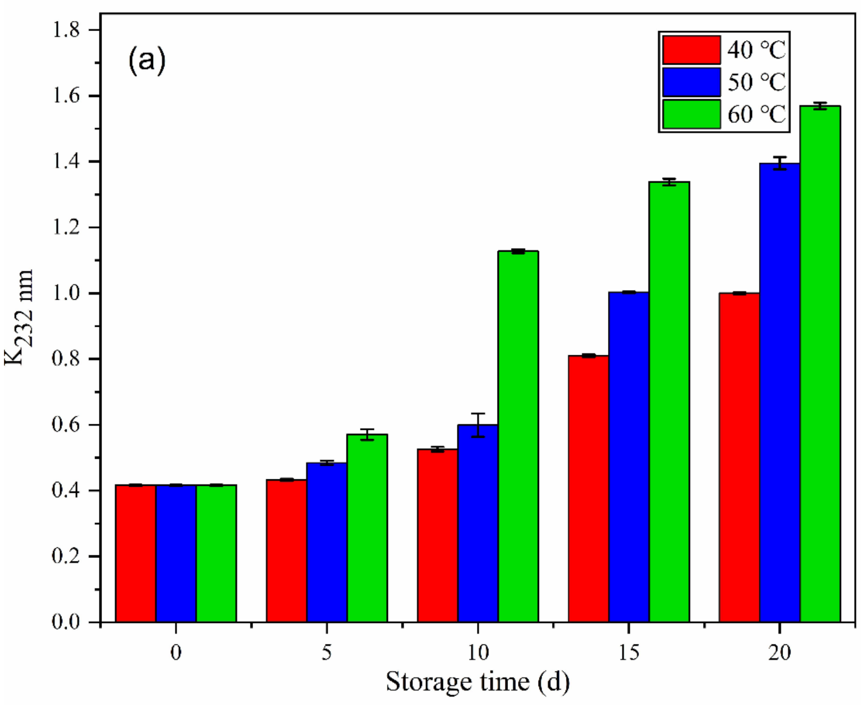

33

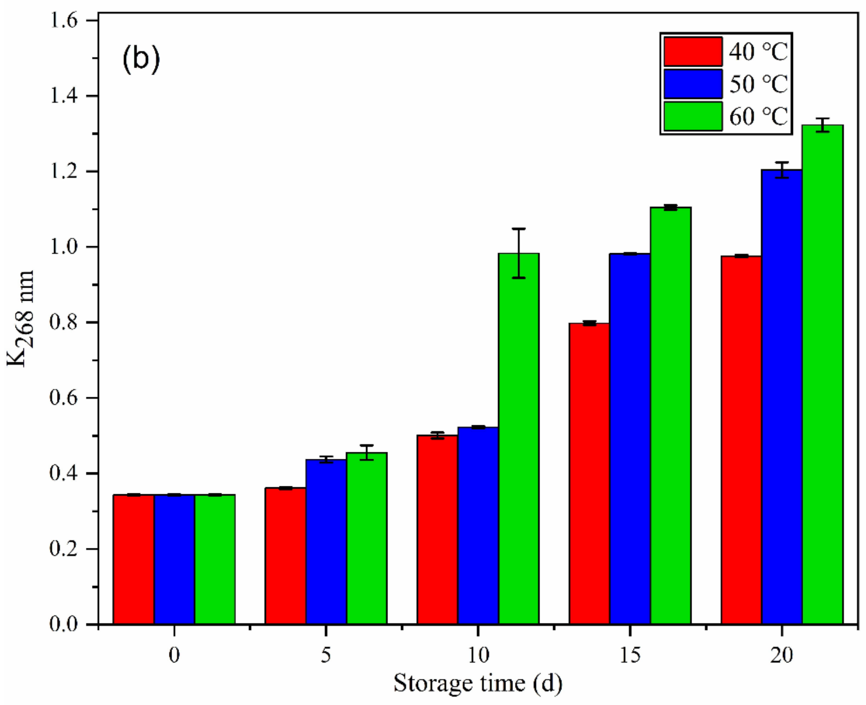
(b)
40 °C
50 °C
60 °C
$K_{268\ nm}$
1.6
1.4
1.2
1.0
0.8
0.6
0.4
0.2
0.0
0
5
10
15
20
Storage time (d)

Table S1. The changes in the peroxide values (PVs) at various temperatures during accelerated storage were determined by fitting the data to kinetic models, and shelf life predictions were achieved using the Arrhenius equation.

Temperatures	Zero-order model	R^2	First-order model	R^2	Arrhenius equation	Predicted shelf life (d)
40 ºC	PV = 0.0040t + 0.3170	0.881	ln(PV) = –0.0007t + 0.0293	0.936		57.39
50 ºC	PV = 0.0062t + 0.3770	0.924	ln(PV) = –0.0009t + 0.0336	0.957	ln(k) = –2267.5/T + 4.0191	44.44
60 ºC	PV = –0.0183t + 0.6315	0.953	ln(PV) = –0.0017t + 0.0501	0.960		23.12
